# A comparison of endoscopic and microscopic inlay butterfly cartilage tympanoplasties and their educational utility

**DOI:** 10.1371/journal.pone.0241152

**Published:** 2020-10-30

**Authors:** Noor Dina Hashim, Se A. Lee, Seung Hyun Jang, In Seok Moon

**Affiliations:** 1 Department of Otorhinolaryngology, Universiti Kebangsaan Malaysia Medical Centre, Kuala Lumpur, Malaysia; 2 Department of Otorhinolaryngology, Yonsei University College of Medicine, Seoul, Korea; 3 Department of Otorhinolaryngology, Soonchunhyang University Bucheon Hospital, Soonchunhyang University School of Medicine, Bucheon, Korea; Sapienza University of Rome, ITALY

## Abstract

**Objectives:**

Inlay butterfly cartilage tympanoplasty (IBCT) is a simple grafting technique. Endoscopy facilitates visualization by eliminating blind spots. We analyzed the outcomes of IBCT using both endoscopic and microscopic approaches, and assessed how trainees perceived the educational opportunities afforded.

**Materials and methods:**

Sixty patients who underwent IBCT were allocated to Group I (n = 30; microscopic IBCT) and Group II (n = 30; endoscopic IBCT) by the dates of their visits. Anatomical success was defined as an intact, repaired tympanic membrane; functional success was defined as a significant decrease in the air–bone gap. Postoperative discomfort was analyzed using a visual analog scale (VAS). Thirteen trainees completed structured questionnaires exploring anatomical identification and the surgical steps.

**Results:**

The surgical success rates were 96.7% in Group I and 100% in Group II. We found no between-group differences in the mean decrease in the air–bone gap or the extent of postoperative discomfort. Significant postoperative hearing improvements were evident in both groups. The mean operative time was shorter when the microscopic approach was chosen (17.7±4.53 vs. 26.13±9.94 min). The two approaches significantly differed in terms of the identification of external and middle ear anatomical features by the trainees, and their understanding of the surgical steps.

**Conclusion:**

Both endoscopic and microscopic IBCT were associated with good success rates. The endoscopic approach facilitates visualization, and a better understanding of the middle ear anatomy and the required surgical steps and thus is of greater educational utility.

## Introduction

Myringoplasty is a common ontological procedure; many techniques and types of grafts are available. It is simple to harvest tragal cartilage for an inlay graft, reducing the surgical time and ensuring firm repair. Inlay butterfly cartilage tympanoplasty (IBCT) was first introduced by Eavey in 1998 [1]; children received grafts shaped to resemble butterfly wings. The refreshed edge of the tympanic membrane is inserted into a groove created in the cartilage. As cartilage graft preparation is easy, the entire procedure is shortened. In addition, there is no need to elevate a tympanomeatal flap [2].

In recent years, endoscopy has become incorporated into many otological procedures, affording better visualization of spaces that are difficult to view microscopically. The ear canal is (naturally) not aligned; certain areas (particularly the anterior part of the tympanic membrane) may be obscured by an angulated ear canal or a hump during microscopically guided surgery. Endoscope affords close (and simple) surveillance of this area and related structures. Angled scopes further improve the views, particularly of the middle-ear space [3–6]. However, the classic microscopic approach has not been forsaken. The two-handed instrumental approach is more natural than one-handed surgery; in addition, the microscopic approach affords three-dimensional images whereas endoscopes do not. Both approaches offer benefits that may enhance the interest and skills of otology trainees [7]. Here, we share our observations on the outcomes of endoscopic and microscopic IBTC, and the educational utility of the approaches as viewed by otology trainees.

## Materials and methods

### Patient selection

Sixty patients who underwent butterfly cartilage inlay myringoplasty in the Otorhinolaryngology Department of Severance Hospital from September 2016 to September 2019 were enrolled in this retrospective study. All had small to moderate central perforations that had developed after chronic ear discharge; all perforations had been dry for at least 3 months. Patients who presented from September 2016 to August 2017 underwent microscopic IBCT (Group I, n = 30); those who presented from September 2018 to September 2019 underwent endoscopic IBCT (Group II, n = 30). All underwent initial temporal bone computed tomography for evaluation of the mastoid and middle ear. Prior to clinical examination, a detail explanation of the procedure, indication, options of treatment and potential complications were informed to all patients. A written consent was obtained prior to surgery. With regards to the study, patients who fulfilled the inclusion criteria were consulted through phone calls for permission to use their surgical outcomes as the study data. Verbal consents were documented and subsequently, their data were recruited. Ethical review (4-2013-0642) was obtained from Yonsei University Health System, Institutional Review Board which stated that a written informed consent for a retrospective study is not required.

### Outcome measurements

Anatomical success was defined as an intact, repaired tympanic membrane evident at the 9-month postoperative review. The pre- and postoperative air–bone gap frequencies were recorded at 500 Hz and 1, 2, and 4 kHz. A decrease in the postoperative air–bone gap reflects functional improvement. Surgical times were recorded. Patient postoperative discomfort was measured using a visual analog scale (VAS).

### Surgical procedures

**Fig 1:**
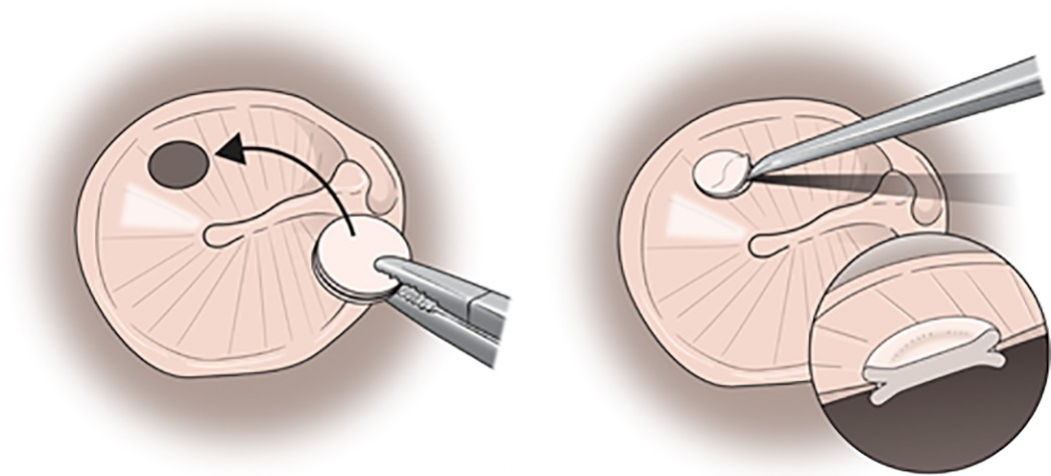
The concept of butterfly cartilage inlay myringoplasty. (Left) A groove is created at the center of tragal cartilage to create a butterfly-shaped graft. (Right) The butterfly graft is inserted into the refashioned edge of the tympanic membrane using a pick.

**Fig 2:**
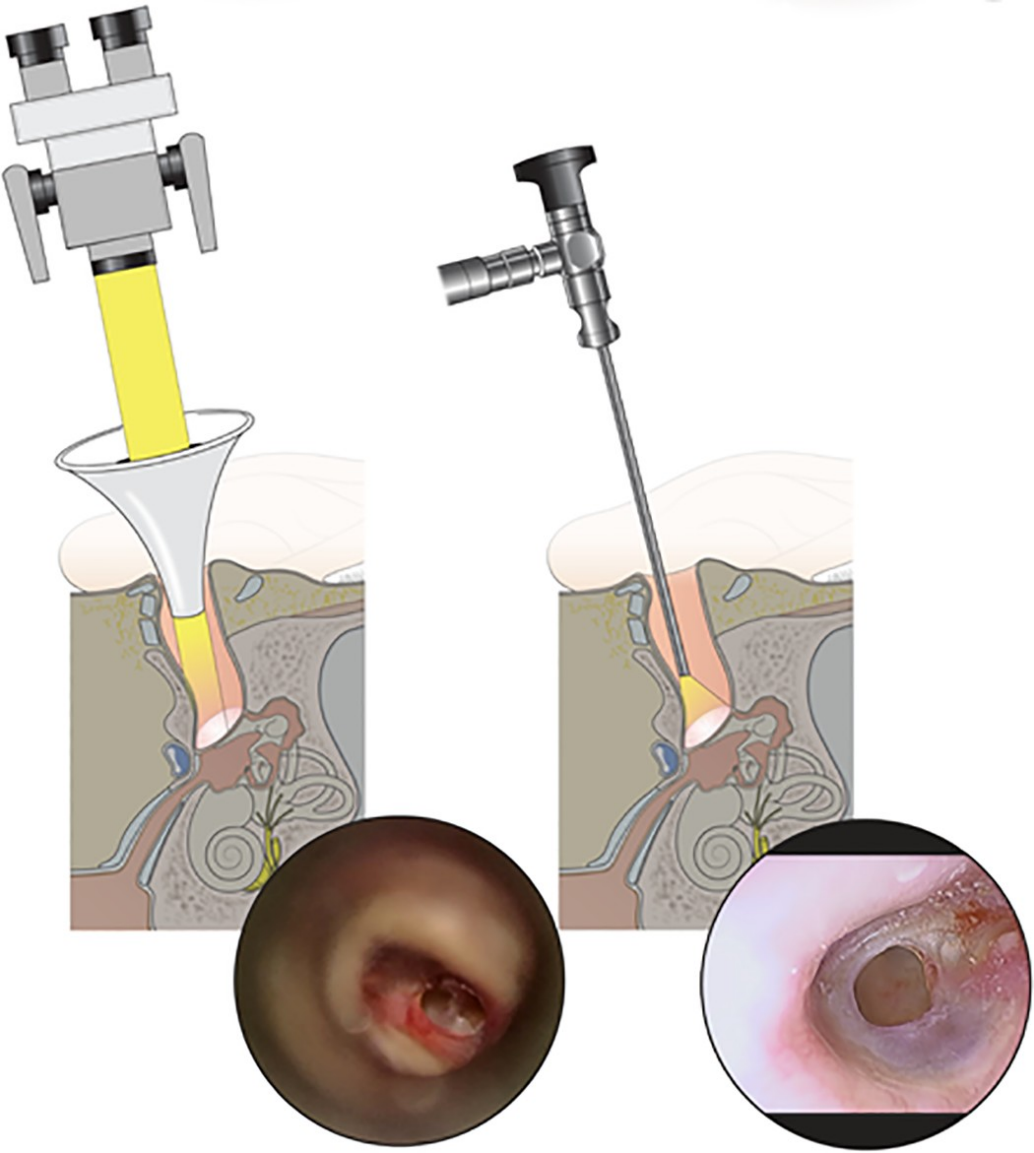
Comparison of microscopic & endoscopic IBCT view via transmeatal approach. (left) Microscopic visualization of tympanic membrane with anterior inferior perforation showing limited anterior viewing of the perforated edge in comparison with (right) endoscopic visualization showing a clear, close-up viewing of the whole circumference of the perforated tympanic membrane.

After the surgical area was sterilized, lidocaine with epinephrine (1:100,000 v/v) was infiltrated at 6 and 12 o’clock lateral to the bony-cartilaginous junction and the tragal area. The margin of the perforated tympanic membrane was refreshed. Tragal cartilage was harvested and carefully trimmed to a diameter 2 mm larger than the perforation. Then a 1–2 mm groove was created around the edge, forming the “butterfly” shape. The perichondrium was removed from one surface. Then the butterfly graft was inserted; the refreshed margin was slipped into the groove using an inlay technique (Fig 1). All operations were performed by a senior surgeon using the transmeatal approach; all patients were under local anesthesia. All patients were discharged on the same day. In all, 30 underwent microscopic procedures (Carl Zeiss, Oberkochen, Germany; Group I) and the other 30 endoscopic procedures (employing a 0°, 3 mm diameter, 14 cm long rigid endoscope and a high-definition monitor and camera (Karl Storz, Tuttlingen, Germany; Group II) (Fig 2 and S1 and S2 Videos).

### Educational utilities

Next, we assessed the educational utility of endoscopic and microscopic IBCT (from the perspectives of trainees). A two-part questionnaire was given to 13 trainees (residents and fellows in otolaryngology). All data were anonymized. Part I consisted of four questions on middle ear anatomy, and four on the surgical steps of endoscopic and microscopic IBCT; the possible responses were “no,” “partial,” or “total” identification and understanding. Each “total” identification or understanding was scored 2, each “partial” response was scored 1, and each “no” response was scored 0. Part II assessed the extents of educational satisfaction associated with the two approaches; the possible answers were “yes,” “no,” and “I am not sure or do not know.”

### Statistical analyses

The Mann-Whitney U-test was employed to compare the functional success and discomfort rates. The questionnaire data were first descriptively analyzed, and then the identifications of anatomical features and surgical steps explored in Part I were scored. The Student t-test was used to compare endoscopic and microscopic data. A p-value < 0.05 was considered statistically significant. All analyses were performed using SSPS ver. 20.0 software.

## Results

The demographic data and surgical outcomes are summarized in Table 1. The graft uptake rates were 96.7% in Group I and 100% in Group II. Neither the mean pre-/postoperative air–bone gaps (ABG) nor the mean air–bone gap decreases differed between the groups (all p>0.05). Group I surgery required a mean of 17.7±4.53 min and Group II surgery required 26.13±9.94 min (p = 0.018). The postoperative discomfort scores were similar in the two groups (3.06±0.87 vs. 2.93±0.75) (p = 0.681).

**Table 1:** Demographic data and outcomes of microscopic (Group I) vs endoscopic (Group II) IBCT.

	Group I	Group II	p-value
**Demographics**			
Number of patients	30	30	
Gender (male:female)	10:20	9:21	
Mean age (years)	60.77±17.33	55.11±13.63	
Site (right: left)	16:14	15:15	
Size of perforation (small:medium:large)	4:12:1	5:10:1	
Mean follow up duration (months)	15.63±11.52	9.53±9.53	
Preoperative air-bone gap (dBHL)	18.68± 6.5	17.19±6.47	0.528
**Outcomes**			
Anatomical Success	100%	96.7%	
Postoperative air-bone gap (dBHL)	13.24±6.75	9.38±6.04	0.104
Functional Improvement (dBHL)	6.48±4.51	9.08±5.65	0.167
Perioperative discomfort (VAS score)	3.05 ± 0.87	2.94 ± 0.75	0.681

Of the 13 trainees who responded to the questionnaire, most reported that the endoscopic approach allowed better identification of anatomical features and a better understanding of the surgical steps (Table 2). Most admired the advantages of the endoscopic approach although 53.8% reported that it was challenging; 61.5% believed that the one-handed approach was a disadvantage (Table 3).

**Table 2:** Educational evaluation between microscopic vs endoscopic approached (n = 13).

	Microscopic approach	Endoscopic approach	p-value
Identification of external and middle ear anatomy	6.62 ± 1.39	7.23 ± 1.80	0.358
Understanding of surgical steps	6.53 ± 1.42	7.45 ± 1.33	0.285
Total scores	13.15 ± 2.62	14.46 ± 3.08	0.284
Scores in senior trainees (n = 8)	13.15 ± 2.62	14.46 ± 3.08	0.284

**Table 3:** Questionnaire regarding identification of anatomy and surgical steps and educational satisfaction.

**PART I: UNDERSTANDING OF IDENTIFICATION ON EXTERNAL/MIDDLE EAR ANATOMY AND SURGICAL STEPS IN IBCT**	**ENDOSCOPIC APPROACH**			**MICROSCOPIC APPROACH**		
**Identification of anatomy**	No	Partial	Total	No	Partial	Total
Have you identified the tympanic membrane?	-	1(7.7%)	12(92.3%)		4(30.8%)	9(69.2%)
Have you identified the tympanic annulus?	1(7.7%)	1(7.7%)	11(84.6%)		7(53.8%)	6(46.2%)
Have you identified the handle of malleous?	-	1(7.7%)	12(92.3%)		3(23.1%)	10(76.9%)
Have you identified middle ear structures including promontory, RW and tympanic segment of facial nerve?	-	1(7.7%)	12(92.3%)		7(53.8%)	5(46.2%)
**Understanding of surgical steps**	No	Partial	Total	No	Partial	Total
Instillation of local anaesthesia at 6 and 12 o’clock of ear canal	-	1(7.7%)	12(92.3%)		1(7.7%)	12(92.3%)
Examination of perforation margin especially the anterior part	-	1(7.7%)	12(92.3%)	1(7.7%)	6(46.2%)	6(46.2%)
Refashioning of the perforation margin	-	1(7.7%)	12(92.3%)		3(23.1%)	10(76.9%)
Insertion of butterfly cartilage tympanoplasty using inlay technique	-	3(23.1%)	10(76.9%)		8(61.5%)	5(38.5%)

**PART II: ENDOSCOPIC VS MICROSCOPIC APPROACH EDUCATIONAL SATISFACTION**	**Yes**	**No**	**Not sure/do not know**
Can you identify the tympanic membrane and middle ear structures better in endoscopic approach?	13(100%)		
Can you understand the surgical steps better in the endoscopic approach compared to microscopic approach?	12(92.3%)	1(7.7%)	
Do you think that the endoscope provides a better view of the surgical field?	13(100%)		
Do you think that the endoscope is offer better information than microscope for education purposes?	12(92.3%)	1(7.7%)	
Do you think that endoscopic surgery is more challenging to perform than microscopic surgery?	7(53.8%)	5(38.5%)	1(7.7%)
Do you think that the one-hand technique in endoscopic approach is a disadvantage compared to two-hands free microscopic approach?	8(61.5%)	4(30.8%)	1(7.7%)
Do you think that the type of surgical approach may influence the operative time?	7(69.2%)	1(7.7%)	3(23.1%)
Do you think that to learn the techniques in endoscopic surgery are more difficult than microscopic surgery?	3(23.1%)	6(46.2%)	4(30.8%)
Would you like to learn endoscopic technique as part of your training?	12(92.3%)		1(7.7%)

## Discussion

Many techniques and graft types have been used to rapidly and successfully repair perforations without compromising hearing. IBCT is fast and the associated harvesting is simple [1–3]. Only local anesthesia is required, postoperative pain is minimal, and the patient can be discharged on the same day. The technique is reliable and there is no need for elevation of a tympanomeatal flap. The outcomes may be even better than those of conventional tympanoplasty [4]. Although Neto *et al*. expressed concern that the natural rigidity of cartilage might render it unsuitable as graft material, subsequent studies have revealed no differences in postoperative hearing gains when fascia, perichondrium, or cartilage was grafted [2]. In our study, the graft uptake rates were high in both Groups I and II (94.1% and 100%).

The endoscopic and microscopic approaches are mutually complementary. The endoscopic approach affords close panoramic views of normally hidden areas such as the anterior part of the annulus (which is often obscured by a tortuous ear canal or a hump, compromising graft uptake) [8]. Endoscopic IBCT optimally treats large perforations [9]. Nevertheless, a one-handed, endoscopic transmeatal approach may be challenging. The microscopic approach has the advantages of magnification and light filters, but affords only a limited view of the ear canal, as some areas are hidden. However, the approach is two-handed and the views feature depth. We found that microscopic IBCT was shorter than endoscopic treatment. This is surprising, as many previous studies have found otherwise [10,11]. At the commencement of our study, a trained surgeon began to use the endoscopic technique. Dogan and Bayraktar [8] reported that mastering endoscopic tympanoplasty required about 60 operations, associated with a gradual decrease in operative time with maintenance of the graft uptake success rate and the hearing results. Understandably, the transition period features a learning curve.

Randomization by visit timing may have caused bias, although all subjects met the inclusion criteria. However, the senior author commenced endoscopic surgery only in September 2018; patients he treated earlier via microscopic surgery have been included in this study. This is the best way to reduce bias.

We explored whether the endoscopic technique was associated with less postoperative discomfort than the microscopic approach. Kakehata *et al*. [12] reported less postoperative pain and a lower requirement for painkillers in patients who underwent endoscopic (compared to microscopic) middle-ear surgery, attributed to the fact that the endoscopic approach does not require skin incision or extensive bone removal. Endoscopic IBCT affords significant advantages when treating pediatric cases; postoperative discomfort is low and the surgical success rate good [13,14]. However, we found no significant between-group difference in postoperative discomfort. In terms of hearing gain, the mean pre- and postoperative air–bone gaps of the groups did not differ, and both improved significantly after operation. It is logical that an intact, repaired membrane will conduct sound well; the surgical approach is irrelevant.

The educational data were interesting. The respondents included eight senior trainees (with us for over 4 years); the remaining five were in year 2 of residency. Almost all stated that the endoscopic approach gave them a better grasp of anatomical structures and the surgical steps. Using the microscopic approach, some could identify only some structures and understand only certain surgical steps. Notably, although over half found that the one-handed endoscopic approach was technically challenging, most planned to use this approach in their future careers. The responses also varied by years of experience. Senior trainees familiar with ear anatomy (because of prior microscopic training) could describe the differences between the two techniques. They considered that the endoscopic approach “simplified” surgery, but that it required comprehensive training. Giannicola *et al*. [5] also reported that the endoscopic technique was educationally better. Both the conventional microscopic and the endoscopic approach must be learned step-by-step to build confidence and competence.

## Conclusion

Both endoscopic and microscopic IBCT were associated with good outcomes. The choice of approach reflects individual preference. However, the endoscopic technique allows trainees to better understand the middle ear anatomy and the surgical steps. This must be considered when a training surgeon selects a technique.

## Supporting information

S1 Video Surgical video of microscopic IBCT. (MP4)

S2 Video Surgical video of endoscopic IBCT. (AVI)

S1 Questionnaire Questionnaire on identification of anatomy, surgical steps and educational satisfaction. (PDF)
