# Supplementary material for: A comparison of endoscopic and microscopic inlay butterfly cartilage tympanoplasties and their educational utility
Source: PLoS One. 2020 Oct 30;15(10):e0241152. doi: 10.1371/journal.pone.0241152 (PMC7598459; doi:10.1371/journal.pone.0241152)
Supplement: S1 Questionnaire — (PDF) [file pone.0241152.s003.pdf]

QUESTIONNAIRE ON UNDERSTANDING AND EDUCATIONAL SATISFACTION AMONG MEDICAL PERSONNEL ON ENDOSCOPIC VS MICROSCOPIC - APPROACHED SURGERY

A) DEMOGRAPHIC

1) AGE : ☐ 20-30 ☐ 31 – 40 ☐ 41 – 50

2) GENDER : ☐ MALE ☐ FEMALE

3) CURRENT POSITION : RESIDENT ☐ YEAR__________

FELLOW ☐

4) ROLE

1) RESIDENT :

surgeon ☐ assistant ☐

2) FELLOW

surgeon ☐ assistant ☐

5) EXPOSURE / EXPERIENCE

i) TECHNIQUES

Endoscopic surgery ☐ Microscopic surgery ☐ Both ☐

ii) YEARS OF EXPOSURE / EXPERIENCE:

METHODS	<2 years	2-4 Years	>4 years
Microscopic surgery			
Endoscopic surgery			

B) **UNDERSTANDING OF IDENTIFICATION ON EXTERNAL/MIDDLE EAR ANATOMY AND SURGICAL STEPS IN BCIM**

1) IDENTIFICATION OF EXTERNAL/MIDDLE EAR ANATOMY	Endoscopic approach understanding			Microscopic approach understanding		
	No	Partial	Total	No	Partial	Total
Have you identified the tympanic membrane and its related structures?						
Have you identified the tympanic annulus?						
Have you identified the handle of malleous?						
Have you identified middle ear structures including promontory, round window and tympanic segment of facial nerve?						
2) UNDERSTANDING OF SURGICAL STEPS						
Instillation of local anaesthesia at 6 and 12 o'clock of ear canal						
Examination of perforation margin especially the anterior part						
Refashioning of the perforation margin						
Insertion of butterfly cartilage tympanoplasty using inlay technique						

C) ENDOSCOPIC VS MICROSCOPIC APPROACH EDUCATIONAL SATISFACTION

NO	QUESTIONS	Yes	No	Not sure/ do not know
1	Did you identify the tympanic membrane and its related structures better in endoscopic approach?			
2	Did you identify the surgical steps and technique better in the endoscopic approach compared to microscopic approach?			
3	Do you think that the endoscope provides a better magnification and view of the entire surgical field?			
4	Do you think that the endoscope is better than microscope for education purposes?			
5	Do you think that endoscopic surgery is more difficult to perform than microscopic surgery?			
6	Do you think that the one-handed endoscopic approach is a disadvantage compared to microscopic approach?			
7	Do you think that the choice of the surgical approach may influence the operative time?			
8	Do you think that the learning curve of endoscopic surgery is longer and more difficult than microscopic surgery?			
9	Considering the advantages and disadvantages of endoscopy would you choose this surgical approach in your training?			

SCORE :

Section B1: Total : 2, Partial : 1, No : 0
Marks (0 – 8)

Section B2 : Total : 2, Partial : 1, No : 0
Marks (0 – 8)
